# Combined optical-electromechanical wearable sensors for cardiac health monitoring

**DOI:** 10.1117/1.JBO.30.6.067002

**Published:** 2025-06-11

**Authors:** Michal Katan, Rui M. R. Pinto, Shiran Arol-Wiegand, Bar Atuar, Alon Tzroya, Hamootal Duadi, K. B. Vinayakumar, Dror Fixler

**Affiliations:** a Bar-Ilan University, Faculty of Engineering, Ramat Gan, Israel; b Bar-Ilan University, The Institute of Nanotechnology and Advanced Materials, Ramat Gan, Israel; c International Iberian Nanotechnology Laboratory, Braga, Portugal; d Birla Institute of Technology and Science, Pilani K.K. Goa Campus, Portugal

**Keywords:** optics, wearable devices, absorption, scattering, biosensor, physiological parameters

## Abstract

**Significance:**

Integrating multiple biosensors improves the sensitivity and precision of physiological measurements in healthcare monitoring. By combining sensors that target different physiological parameters, a more comprehensive assessment of a subject’s health can be achieved.

**Aim:**

We evaluate the performance of two biosensors for extracting cardiac parameters: a textile-based strain sensor for measuring respiratory rate and an optical sensor for measuring heart rate, ${\mathrm{SpO}}_{2}$, and respiratory rate. The objective is to determine optimal placement conditions for each sensor and assess their feasibility for integration into a single wearable system.

**Approach:**

Two experimental setups were tested. In the first, the strain sensor was placed on the subject’s shirt, while the optical sensor was positioned on the external wrist. In the second, both sensors were placed on the chest, under the shirt. The accuracy and performance of each sensor were analyzed in both configurations.

**Results:**

The optical sensor demonstrated improved accuracy when placed on the chest compared to the wrist, whereas the strain sensor provided similar results for both configurations.

**Conclusions:**

We demonstrate that sensor placement significantly affects measurement quality, emphasizing the importance of optimizing placement when integrating multiple biosensors. Future work will focus on developing a unified wearable system that leverages the strengths of both sensors for comprehensive physiological monitoring.

## 1 Introduction

Integration of multiple biosensors plays an important role in various fields, ranging from healthcare to environmental monitoring. By combining different biosensors, each designed to target specific conditions or parameters, the overall sensitivity, precision, and reliability of the measurement process can be significantly improved. These advanced biosensors, often integrated into wearable devices or implantable systems, provide real-time and continuous data on crucial cardiac functions such as heart rate (HR), respiratory rate (RR), oxygen saturation (${\mathrm{SpO}}_{2}$), and blood pressure, offering insights into an individual’s cardiovascular health and enabling early detection of potential health conditions.^1^

Different types of biosensors are employed for measuring cardiac parameters, each designed to focus on specific aspects of cardiovascular health. Electrochemical biosensors measure electrical changes associated with cardiac biomarkers, such as proteins or enzymes related to heart functions.^2^ Acoustic sensors measure sound waves associated with cardiac activity and can capture heart sounds and provide information about valve functionality.^3^ Optical biosensors use light to detect changes in blood. For instance, photoplethysmographic (PPG) sensors measure variations in light absorption due to volumetric and spectral changes in peripheral blood circulation to determine HR and ${\mathrm{SpO}}_{2}$.^4^^–^^6^ Electromechanical biosensors detect mechanical changes (such as stress or strain) of heart activity; piezoelectric sensors, for example, generate electrical signals in response to mechanical stress, allowing for the measurement of subtle movements or vibrations associated with cardiac activity.^7^

**Fig. 1:**
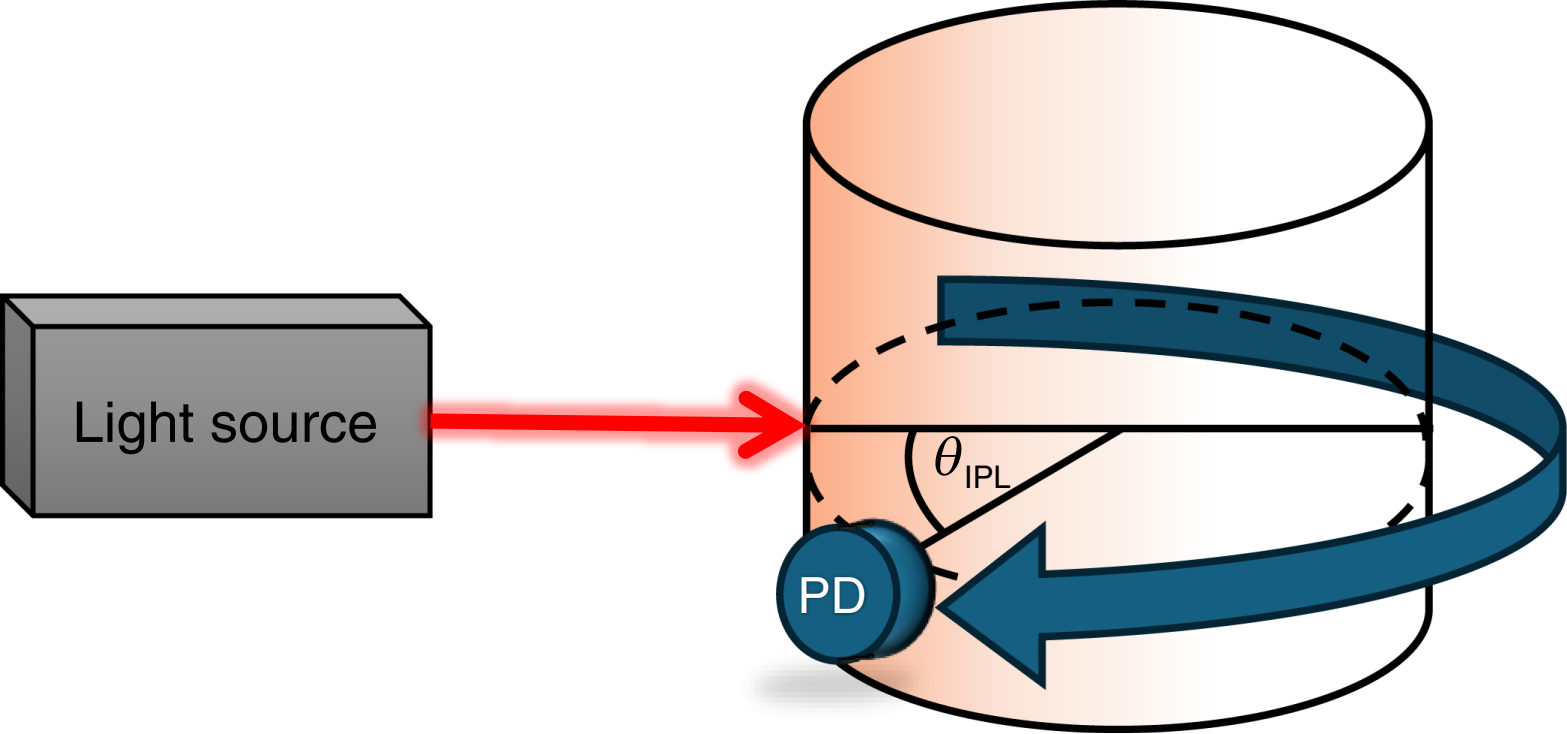
Illustration of the optical system in which the IPL point is measured. A light source illuminates a cylindrical sample, and a photodetector (PD) rotates around the sample collecting light intensity.

Optical biosensors typically rely on the measurement of reflected or transmitted light intensity from tissues. Changes in light intensity can be detected from PPG signals, which represent changes in blood volume. PPG signals can provide vital information on the patient’s cardiac situation, such as HR and RR. Blood is known to be both scattering and absorptive, yet its absorption is about 10 times stronger than its scattering.^8^ Therefore, ideally, optical methods for measuring biological tissues would focus on sensing changes in absorption rather than just measuring the reemitted light intensity. Unfortunately, the light intensity reemitted from tissues is dependent on the scattering and on the absorption, and both properties are inseparable. Hence, detecting more complex variables, such as ${\mathrm{SpO}}_{2}$, requires using multiple light sources for assessing the absorption. These methods measure the ratio of light intensity from different light sources, which results in inherent errors due to scattering changes and requires an external calibration.^9^^,^^10^ To overcome these challenges, we designed an optic biosensor for measuring cardiac parameters, using a physical property called the iso-pathlength (IPL) point.^11^^,^^12^ The IPL point is a specific angle in accordance with the light source (Fig. 1), in which the light intensity is not affected by the scattering and the absorption can be extracted.^13^^–^^16^ The IPL point provides self-calibration, from which cardiac parameters can be extracted accurately. Using the IPL point’s principles, the designed optical biosensor is suitable for extracting cardiac parameters, such as HR, RR, and ${\mathrm{SpO}}_{2}$.

Optical measurements of RR are highly affected by the significant motions of the chest; therefore, in this work, we combine the optical biosensor with another one. The other type of device used in this work is a textile-based strain sensor, a resistive sensor that responds to the stretching in the longitudinal direction. The textile strain sensor was placed on the chest area to sense the expansion and contraction of the chest with each breath. This motion is translated into an electric signal, and the RR can be extracted.

In this paper, we present the combination of these two devices: the self-calibrated optical sensor, produced by Bar-Ilan University (BIU), and the flexible strain sensor, produced by International Iberian Nanotechnology Laboratory (INL). The sensors were combined to extract and compare RR values, to understand the advantages and limitations of each one in order to integrate them into a single wearable device in the future. The optical biosensor can also extract HR and oxygen saturation, as described in our previous publication.^11^ All measurements were taken in parallel and were compared with those taken with a commercial pulse oximeter, which is the gold standard for HR and ${\mathrm{SpO}}_{2}$ measurements, and to manual respiratory counting, which is the gold standard for RR measurements.

## 2 Materials and Methods

### 2.1 Textile-Based Strain Sensor

**Fig. 2:**
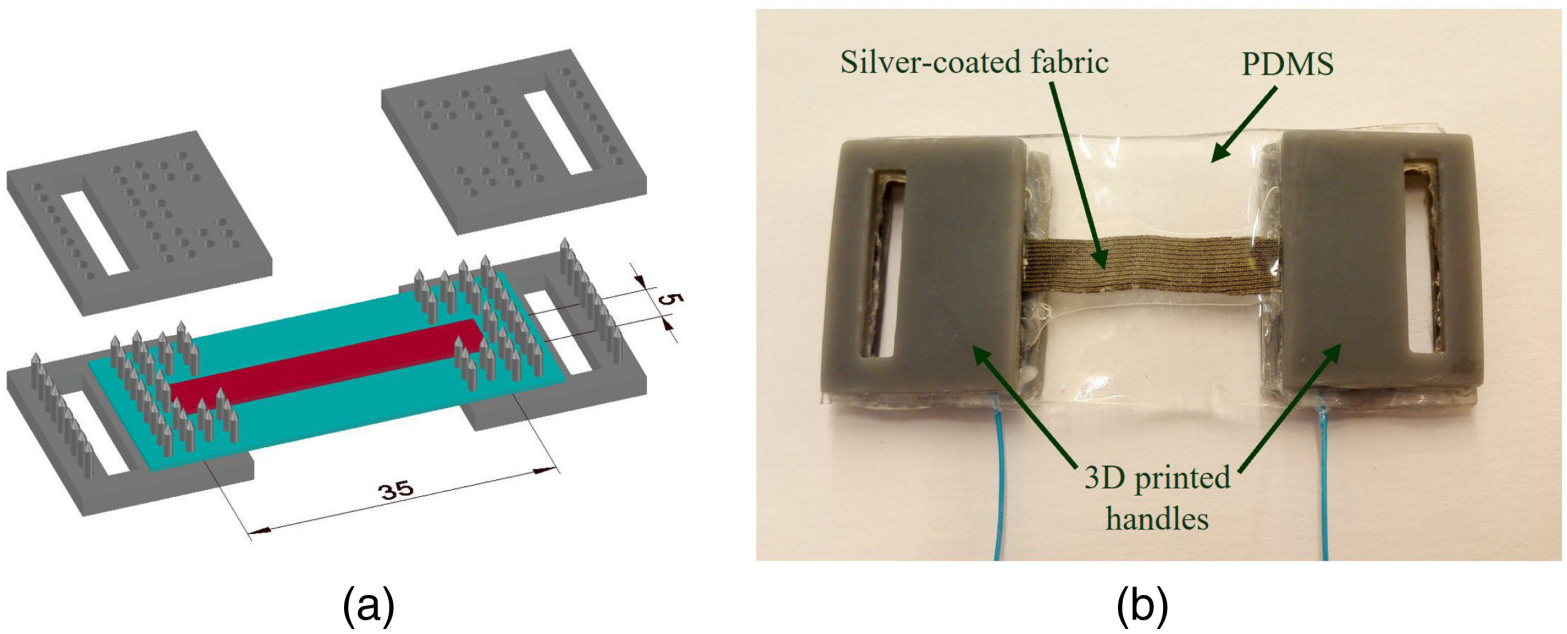
Encapsulated textile strain sensor. (a) A schematic illustration of the strain sensor. (b) Picture of the final strain sensor.

The strain sensor active material is a silver-coated stretchable fabric (Stretch conductive fabric 4900, by Holland Shielding Systems BV, Dordrecht, the Netherlands). The fabric was cut (35×5  mm) and electrically connected to thin, nonobtrusive cables using flexible conductive silver paste (CW2900, by Chemtronics, Kennesaw, Georgia, United States). The sensor was laminated between two 0.5 mm-thick polydimethylsiloxane (PDMS) layers for waterproofing and mechanical stability, protecting the sensitive fabric. PDMS (SYLGARD™ 184 Silicone Elastomer, by Dow Chemical Company, Midland, Michigan, United States) was prepared by mixing the base silicone with the curing agent in the proportion of 10:1 (w/w) and curing at 60°C for 12 h. The sensor was placed between two solid PDMS layers, sealed around with liquid PDMS and cured again. The liquid PDMS did not penetrate the silver-coated textile. Finally, the laminated textile was cut and clamped at the ends using 3D-printed parts (see Fig. 2). The active length of the sensor (between mounts) is 25 mm.

### 2.2 Optical Biosensor

**Fig. 3:**
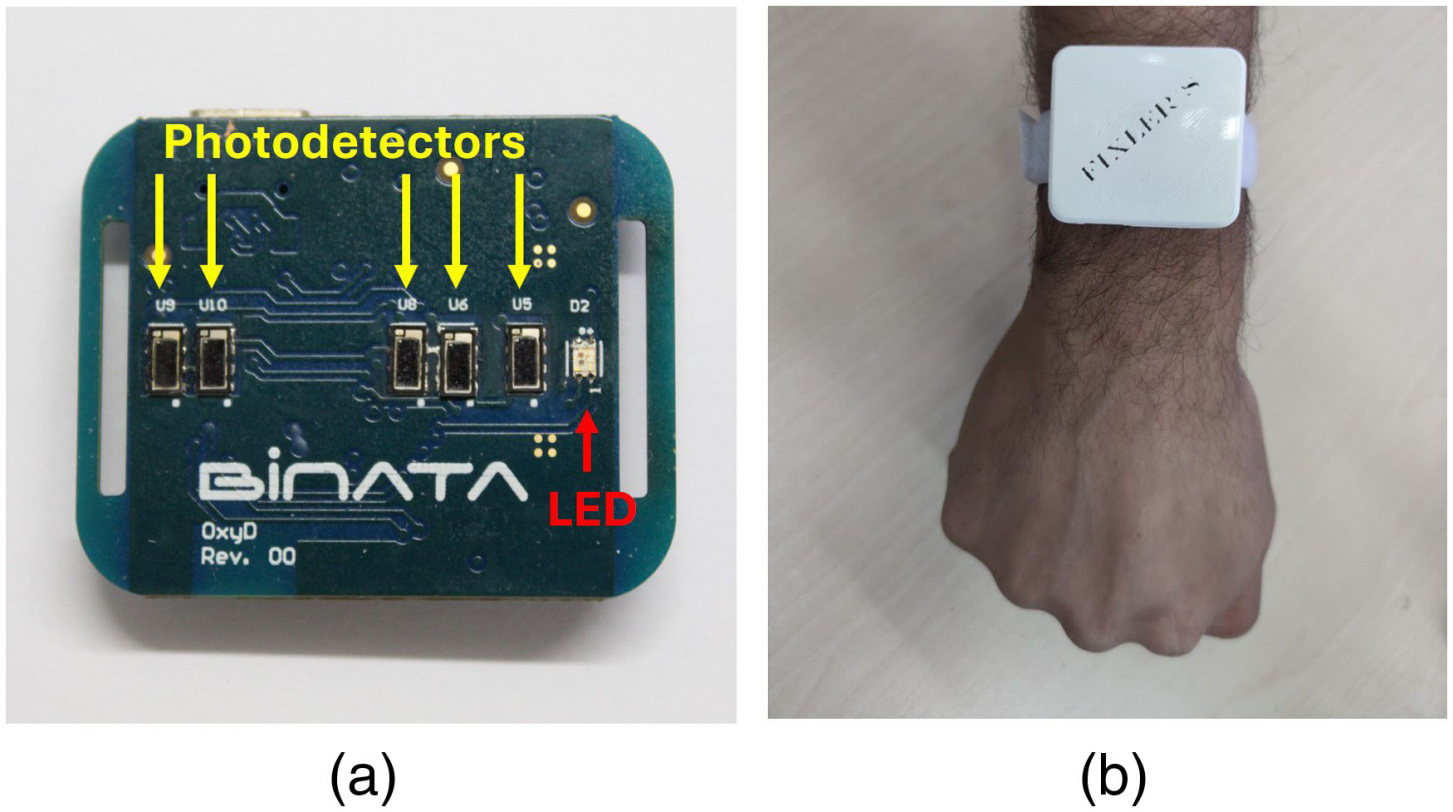
(a) The optical sensor’s printed circuit board, consisting of a single wavelength light source and five PDs. (b) The sensor with its protective case on a subject’s wrist.

The optical biosensor is based on the properties of the IPL point, as discussed in the introduction and detailed in our previous publication.^11^ The biosensor consists of a red LED with a 655 nm wavelength and five photodetectors (PDs), as depicted in Fig. 3. The PDs collect light intensity reemitted from the tissue at a frequency of 100 Hz, to fit the necessary sampling rate of PPG signals.^4^ One of the PDs is in the IPL point’s location in accordance with the LED position, from which the light intensity can be translated to absorption coefficients. By analyzing the light intensity read from the IPL point’s PD, and another PD that serves as a reference, absorption coefficients can be extracted. The reference PDs are determined in accordance with the measurement location, wrist or chest.

### 2.3 Data Acquisition and Analysis

The strain sensor was driven with a current source of 10 mA and a preamplifier with a gain of 70. This current was chosen as it provided a sufficient signal for acquisition. The strain sensor is connected to an oscilloscope (Agilent Technologies, Santa Clara, California, United States, DSO5014A) for data collection, whereas the optical biosensor is run by MATLAB through a universal asynchronous receiver-transmitter (UART) communication protocol. The optical biosensor collects the reemitted light intensity from the tissue, whereas the strain sensor detects stretching motions. After reading the data from both sensors, a postprocessing session was required. By using a Fourier transform, the raw data were translated into the frequency domain, and then both were filtered in the range of 0.14 to 1 Hz to find the RR. Next, the data from the optical sensor was also filtered in the range of 1 to 3 Hz to find the HR.^4^^,^^17^ The extracted values are multiplied by 60 to reach units of beats/breaths per minute (bpm) for HR and RR, respectively. In the next step, we calculated ${\mathrm{SpO}}_{2}$ from the optical sensor’s IPL point and an additional reference PD, by translating the light intensity to absorption coefficients of the PPG signal. The PPG waveform has both alternating and constant components, denoted as AC and DC, whereas the ratio between them produces a signal proportional to the arterial blood, from which ${\mathrm{SpO}}_{2}$ can be extracted.^11^

### 2.4 Human Experiments

In this work, we designed experiments from which we could compare the results and determine the most accurate position and use for each one of the sensors. The optical biosensor is usually placed on the wrist, whereas the strain sensor should be placed on a stretchable material such as a shirt. Therefore, first, we placed them in their optimal and traditional locations and tested 15 subjects with the optic biosensor placed on the wrist, while the strain sensor was attached to the shirt. In the next experiment, we tested subjects with both sensors placed on the chest. The second experiment also included testing 15 subjects, a few of whom participated in both experiments. The sensors were attached to an elastic band, which was wrapped around the subject’s chest, under the shirt. For these experiments, we developed a MATLAB code that operates them simultaneously, with each measurement lasting 50 s. In both experiments, all subjects were asked to count the number of breaths that they took during the measurement, as an RR reference, and wear a pulse oximeter (CMS50M, Contec Medical Systems, Qinhuangdao, China) for HR and ${\mathrm{SpO}}_{2}$ reference. Note that the extracted values of HR and RR have units of beats per minute (as explained in the section on data analysis), although each experiment lasted only 50 s. For this reason, the references for RR have been multiplied by 1.2 to reach the correct units.

Tables S1 and S2 in the Supplemental Material describe the demographic data of the subjects; Tables S3 and S4 in the Supplemental Material depict the results.

## 3 Results

### 3.1 Optical Sensor on Wrist, Strain Sensor on Chest

**Fig. 4:**
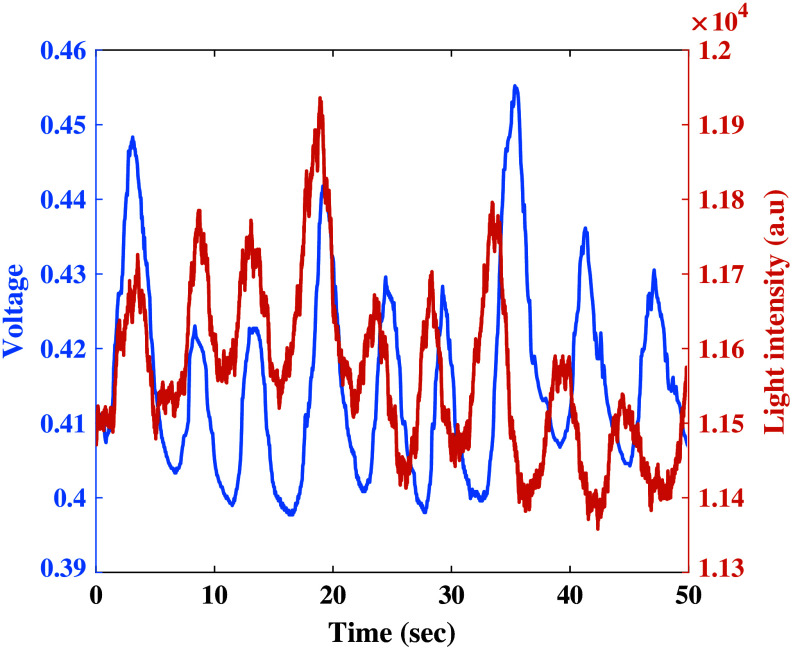
Raw data of respiratory rate signals as a function of time. The blue plot represents the voltage measured by the strain sensor, whereas the orange plot represents the light intensity measured by the optical sensor. The subject counted nine breaths during the measurement, corresponding to the nine peaks shown in the measurement of both sensors. This reference RR is translated to 10.8 bpm. The calculated RR is 12 bpm for the optical sensor and 10.8 bpm for the strain sensor.

**Fig. 5:**
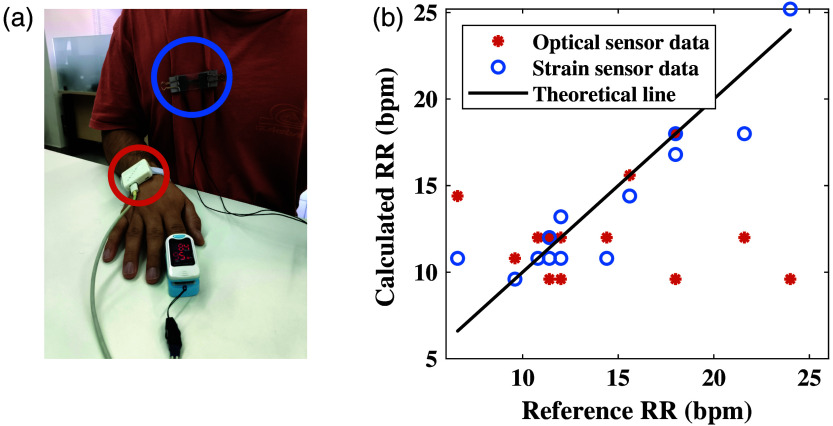
(a) The optical sensor is placed on the external side of the wrist with the strain sensor attached to the shirt on the chest. A pulse oximeter is used on the finger as a reference device. (b) RR results of the first experiment of both optical and strain sensors, compared with the reference measurements. The linear line represents the reference measurements, whereas the marks represent the extracted values from the sensors.

In the first set of experiments, the optical sensor was mounted on the wrist, whereas the strain sensor was installed on the shirt of the subjects. Figure 4 shows the signals of both sensors as a function of time, with the large peaks representing the subjects’ respiration. Although the two signals appear to be unsynchronized, they can be calibrated to ensure proper alignment. Figure 5 depicts the RR results for both sensors, with the subjects’ counted breaths used as reference. The root mean square error (RMSE), calculated by the “fit” command in MATLAB, produced by the optical sensor is 2.68, whereas the RMSE by the strain sensor is 1.92.

**Fig. 6:**
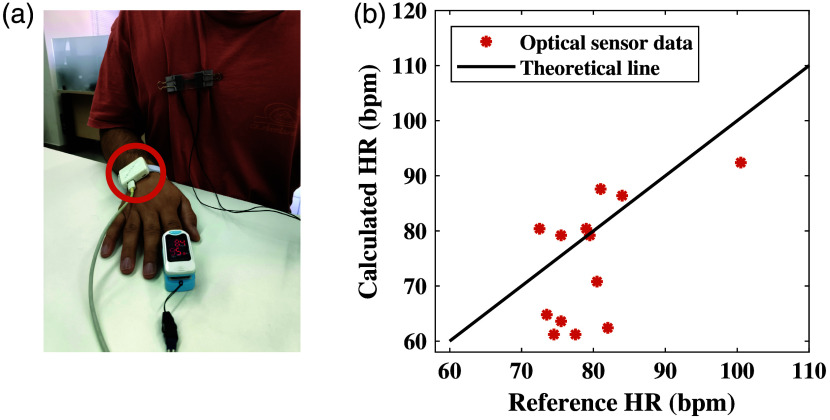
(a) The optical sensor is placed on the external side of the wrist, with a pulse oximeter used on the finger as a reference device. (b) HR results of the first experiment of the optical sensor, compared with the reference measurements. The linear line represents the reference measurements, whereas the marks represent the extracted values from the sensors.

Subsequently, the analysis of the HR data allowed us to compare the optical sensor measurements with a pulse oximeter. The HR results calculated by the optical sensor and the data from the pulse oximeter are plotted in Fig. 6(b). The graph shows a reasonable fit of the optical biosensor.

**Fig. 7:**
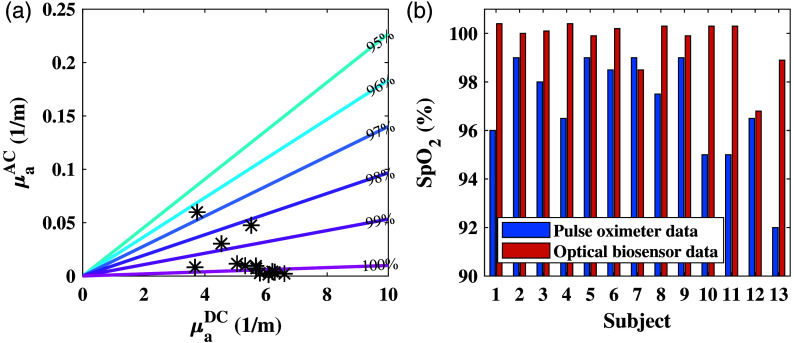
${\mathrm{SpO}}_{2}$ results of the first experiment. (a) AC and DC values of absorption coefficients measured by the IPL point. The asterisks represent the measured values of all 15 subjects, whereas the colored lines represent the theoretical values of 95% to 100% saturation. (b) Comparison between the results of the pulse oximeter (in blue) and the optical biosensor (in orange).

${\mathrm{SpO}}_{2}$ values were extracted from the optical biosensor based on the IPL point’s concept. Figure 7(a) shows the AC and DC results of the absorption measured by the sensor. The colored lines represent the normal range for ${\mathrm{SpO}}_{2}$, calculated from the equation demonstrated in our previous publication,^11^ with ${\mathrm{SpO}}_{2}$ values of 95% to 100%. As anticipated when measuring healthy subjects, most of the measurements were within this range as these are the normal values of oxygen saturation. There were few values that exceed 100%, which is impossible; however, this is within the 0.4% error.^11^ Figure 7(b) shows the comparison of the ${\mathrm{SpO}}_{2}$ values measured by the biosensor, with an average of 99.6%, to the values measured by the pulse oximeter, with an average of 97%.

### 3.2 Sensors Integrated into an Elastic Band, Placed on the Chest

**Fig. 8:**
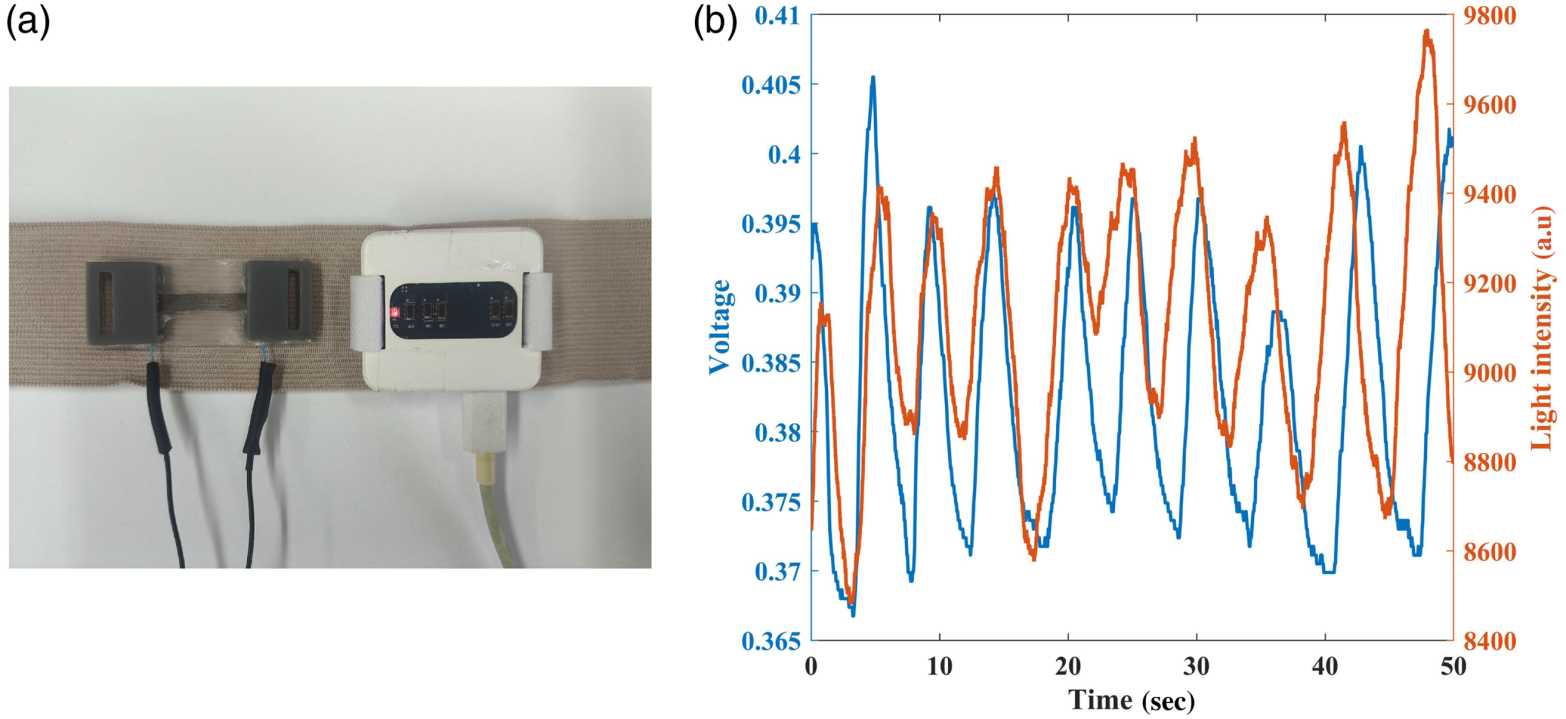
(a) Both sensors are attached to an elastic band, for the second set of experiments, with the strain sensor on the left and the optical sensor on the right. (b) Raw data of respiratory rate measured signals as a function of time. The blue plot represents the voltage measured by the strain sensor, whereas the orange plot represents the light intensity measured by the optical sensor. The subject counted 10 breaths during the measurement, fitting the 10 peaks shown in both sensors. This reference RR is translated to 12 bpm, and the frequency analysis has been calculated as 10.8 bpm by both the optical and the strain sensors.

For this experiment, both sensors were integrated into an elastic band and placed on the chest of the test subjects. As before, we start by analyzing the data respecting the respiratory rate (Fig. 8). The magnitude of the signals obtained with the strain sensor is identical to that of the first experiment. However, the optical sensor signal has fewer high-frequency noise components than before, indicating that this sensor location might be better suited for RR measurements.

**Fig. 9:**
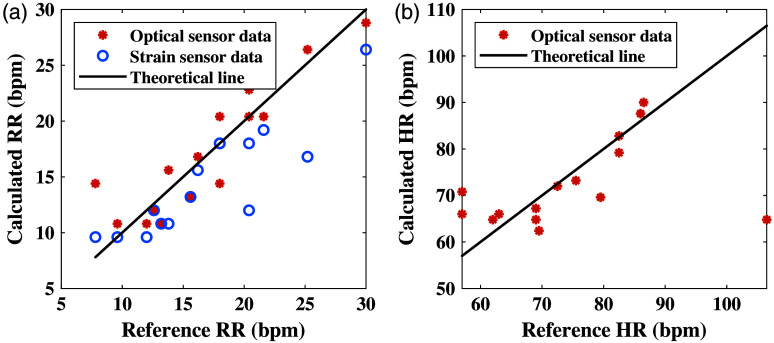
(a) RR results of the second experiment of the optical and strain sensors, both located on the chest, in comparison with the reference measurements. The linear line represents the reference measurements, and the marks represent the extracted values from the sensors. (b) HR results of the second experiment, acquired with the optical sensor, in comparison with the reference measurements. The sensor was placed directly on the chest. The linear line represents the reference measurements, and the marks represent the extracted values from the sensor.

Figure 9 describes the RR and HR results of the optical sensor in comparison with the strain sensor and pulse oximeter. Contrary to our expectations, the strain’s RR results have an RMSE of 2.37, making it higher than our previous experiment. Conversely, the optical sensor shows better results for the RR measurement, with an RMSE of 2.52, in comparison with the previous experiment. This can be explained by the fact that respiration is more visible and stronger on the chest than on the wrist, making it easier to extract RR.

As shown in Fig. 9(b), the HR results have also improved during this session of experiments. The optical sensor produced better results on the chest than on the wrist, with an RMS error of 8.27.

**Fig. 10:**
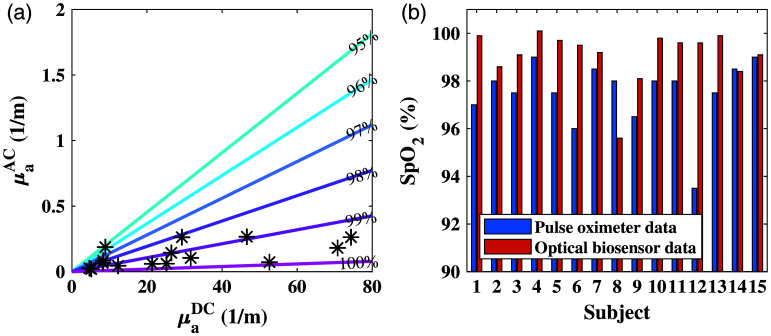
${\mathrm{SpO}}_{2}$ results of the second experiment, with the optical biosensor, are placed on the chest, and the pulse oximeter is placed on the finger. (a) AC and DC values of absorption coefficients measured by the IPL point. The asterisks represent the measured values of ${\mathrm{SpO}}_{2}$ of all 15 subjects, whereas the colored lines represent the theoretical values of 95% to 100% saturation. (b) Comparison between the results of the pulse oximeter (in blue) and the optical biosensor (in orange).

The results of the ${\mathrm{SpO}}_{2}$ measured with the optical sensor are shown in Fig. 10. Figure 10(a) presents the absorption coefficients’ AC and DC components, with all values lying in the range of 95% to 100%. In comparison with the first experiment, the values are more spread out over the DC axis, which can be explained by the location of the measurement. Due to 12 of all 15 subjects being males, with naturally more abundant chest hair, this results in higher values for the DC component of the absorption.^11^ Figure 10(b) demonstrates the comparison of the reference and the calculated values for all subjects. In this case as well, most of the ${\mathrm{SpO}}_{2}$ values extracted from the optical sensor are higher than the reference, with an average value of 99.08% calculated by the optical sensor and an average of 97.5% produced by the pulse oximeter.

## 4 Discussion

In this paper, we have combined strain and optical devices for extracting cardiac parameters from different locations in the human body. The strain sensor is an elastic device that allows its stretching and relaxations to be translated into RR. The optical biosensor’s operation is based on the IPL point’s concept, as reviewed in detail in other papers^11^^,^^18^ as well as covered in this work. The optic sensor is capable of extracting HR, RR, and ${\mathrm{SpO}}_{2}$, allowing for the comparison with the other sensor. Table 1 shows a brief comparison with other noninvasive cardiac health monitoring methods.

**Table 1:** Comparison of other noninvasive cardiac health monitoring methods.

Name	Accuracy				
	Monitoring method	RMSE	MAPE	MAE	References
Garmin	Consumer smartwatch for HR and ${\mathrm{SpO}}_{2}$	3.01 bpm for HR, 3% to 6.7% for ${\mathrm{SpO}}_{2}$	—	2.5% to 5.8% for ${\mathrm{SpO}}_{2}$	19 and 20
Apple Watch Series 7	Consumer smartwatch for blood oxygen saturation estimation	2.9%	—	2.2%	20
TomTom Runner Cardio	PPG-based monitor for HR	—	3.3%	—	21
Hernandez et al.	Estimation of HR and RR from wrist motions	—	—	1.27 bpm for HR and 0.38 bpm for RR	22
Reyes et al.	RR monitor using a smartphone camera	0.414 bpm	—	—	23
Sleepiz One + respiration monitor	Contactless RR monitor device for home use during sleep	—	—	0.39 and 0.48 bpm	24
Withings ScanWatch	Consumer smartwatch for blood oxygen saturation estimation	4%	—	3%	20
**Optical biosensor by BIU**	**PPG-based cardiac parameters monitor**	**0.4% for SpO**_**2**_ **, 8.27 to 9.48 bpm for HR, and 2.52 to 2.68 bpm for RR**	—	—	11, 14, 18, 25
**Strain sensor by INL**	—	**1.92 to 2.37 bpm**	—	—	—

The accuracy detailed here is in terms of root mean square error (RMSE), mean absolute percentage error (MAPE), or mean absolute error (MAE). The last two bolded rows are from the current paper.

In this work, two experiments were executed, in which the main difference was the location of the sensors. In the first experiment, we placed the optical sensor on the external side of the wrist (we proved in previous work that cardiac parameters can be extracted from both sides of the wrist^11^), whereas the strain sensor was placed against the chest, attached to the shirt with clips on both sides. In the second experiment, both sensors were mounted on an elastic band and placed on the chest, directly on the skin.

The RR results of both experiments showed a good fit to the reference of the counted respirations, with the strain sensor showing superiority (smaller error). As for the optical biosensor, the results of the chest were notably better, possibly because the respiration effect is stronger in the chest area, rather than the wrist. The results of the strain sensor were quite similar in both experiments, indicating that the placement does not significantly affect the measurements as long as the sensor is positioned on the chest area. When combining the results from both sensors, the average produces an RMSE of 1.66 for the first experiment, compared with 2.68 for the optical sensor and 1.92 for the strain sensor individually. The results of the second experiment also show improvement, with a combined RMSE of 1.6, compared with 2.52 and 2.37 for the optical and strain sensors, respectively. These results suggest that integrating the outputs of both sensors has the potential to enhance measurement accuracy.

Regarding the HR measurements, the optical biosensor also showed better results on the chest, possibly due to the proximity to the heart. The RMSE of HR results showed an improvement from 9.48 to 8.27. Regarding oxygen saturation, the ${\mathrm{SpO}}_{2}$ measurements of the optical biosensor showed acceptable results by extracting values that are mostly within the range of normal oxygen saturation. In most cases, the optical biosensor produced higher values than the pulse oximeter. Even though the pulse oximeter is the gold standard in this field, the error of the device increases as the oxygen saturation decreases. For this reason, we believe that even in cases where the pulse oximeter showed lower ${\mathrm{SpO}}_{2}$ values, the optical biosensor’s results remain valid.

The present study has several limitations that must be acknowledged to provide a balanced perspective on the findings. First, the sample size of 13 or 15 subjects per experiment is relatively small, limiting the generality of the results. Moreover, the use of different participants across the two experiments introduces potential variability that may affect the comparability of the data. Another significant limitation is the short measurement duration of 50 s, which was chosen due to the technical constraints of the measurement setup. This limited duration may not adequately capture the variability in cardiac parameters and could lead to less reliable results. A longer measurement period, such as 5 min, would likely enhance data robustness and provide a more comprehensive assessment of the sensors’ performance. In addition, the reliance on manual breath counting as the reference for respiratory rate introduces the potential for human error, which could be mitigated in future studies by employing automated systems or clinical-grade reference devices. Despite these limitations, the study highlights the potential for integrating multiple sensors into a wearable system and emphasizes the importance of optimizing sensor placement and combining outputs from different modalities to improve overall accuracy and reliability. Future research should address these limitations by incorporating a larger, more diverse group of subjects, extending measurement durations, and exploring advanced data integration algorithms.

## 5 Conclusion

The findings shown in this work indicate that measuring on the chest leads to the best results. Moreover, the chest location allows the measurement of all three vital parameters, in contrast to the wrist, where RR can be extracted by the optical sensor alone. Measuring with different devices simultaneously has many benefits as they all complement each other, and the advantages of each sensor can hence be utilized. In addition, it is relatively complicated to extract the RR due to the movement of the subject, hence, measuring with two different devices is an advantage and allows for redundancy. The next step of the study is combining the sensors in a single noninvasive and wearable device, on an elastic substrate. For this purpose, the stretchable material of the strain sensor can be utilized, allowing more accuracy in the RR measurements. The optical sensor would complement the former device with its ability to extract multiple parameters in a single measurement, including HR and ${\mathrm{SpO}}_{2}$.

## Supplementary Material

10.1117/1.JBO.30.6.067002.s01

## Data Availability

All data in support of the findings of this paper are available within the paper or as supplemental material.
