# Supplementary material for: Combined optical-electromechanical wearable sensors for cardiac health monitoring
Source: J Biomed Opt. 2025 Jun 11;30(6):067002. doi: 10.1117/1.JBO.30.6.067002 (PMC12152588; doi:10.1117/1.JBO.30.6.067002)
Supplement: Supplementary file 1 [file JBO_030_067002_SD001.docx]

Combined optical-electromechanical wearable sensors for cardiac health monitoring: supplemental document

Table S1. Demographic data of the subjects that participated in the first experiment.

| # Subject | Age | Gender |
| --- | --- | --- |
| 1 | 27 | Female |
| 2 | 29 | Female |
| 3 | 27 | Male |
| 4 | 26 | Female |
| 5 | 28 | Male |
| 6 | 23 | Male |
| 7 | 27 | Male |
| 8 | 25 | Male |
| 9 | 26 | Male |
| 10 | 40 | Female |
| 11 | 26 | Male |
| 12 | 28 | Male |
| 13 | 24 | Male |
| 14 | 27 | Male |
| 15 | 29 | Male |

Table S2. Demographic data of the subjects that participated in the second experiment.

| # Subject | Age | Gender |
| --- | --- | --- |
| 1 | 26 | Female |
| 2 | 25 | Male |
| 3 | 22 | Male |
| 4 | 28 | Male |
| 5 | 27 | Male |
| 6 | 28 | Male |
| 7 | 24 | Male |
| 8 | 24 | Male |
| 9 | 31 | Male |
| 10 | 25 | Male |
| 11 | 31 | Male |
| 12 | 68 | Male |
| 13 | 20 | Male |
| 14 | 27 | Female |
| 15 | 27 | Female |

Table S3. Clinical data of the first experiment.

| # Subject | Reference HR [bpm] | HR extracted by the optical sensor [bpm] | Reference RR [bpm] | RR extracted by the optical sensor [bpm] | RR extracted by the strain sensor [bpm] | Reference SpO2 [%] | SpO2 extracted by the optical sensor [%] |
| --- | --- | --- | --- | --- | --- | --- | --- |
| 1 | 84 | 86.4 | 21.6 | 12 | 18 | 96 | 100.4 |
| 2 | 72.5 | 80.4 | 10.8 | 12 | 10.8 | 99 | 100 |
| 3 | 74.5 | 61.2 | 11.4 | 9.6 | 12 | 98 | 100.1 |
| 4 | 79 | 80.4 | 11.4 | 12 | 10.8 | 96.5 | 100.4 |
| 5 | 77.5 | 61.2 | 18 | 9.6 | 16.8 | 99 | 99.9 |
| 6 | 79.5 | 79.2 | 18 | 18 | 18 | 98.5 | 100.2 |
| 7 | 75.5 | 63.6 | 15.6 | 15.6 | 14.4 | 99 | 98.5 |
| 8 | 81 | 87.6 | 9.6 | 10.8 | 9.6 | 97.5 | 100.3 |
| 9 | 70 | 118.8 | 19.8 | 19.2 | 18 | 96.5 | 99.9 |
| 10 | 73.5 | 64.8 | 24 | 9.6 | 25.2 | 99 | 99.9 |
| 11 | 98 | 61.2 | 12 | 9.6 | 13.2 | 97.5 | 98.8 |
| 12 | 75.5 | 79.2 | 6.6 | 14.4 | 10.8 | 95 | 100.3 |
| 13 | 100.5 | 92.4 | 12 | 12 | 10.8 | 95 | 100.3 |
| 14 | 82 | 62.4 | 12 | 9.6 | 13.2 | 96.5 | 96.8 |
| 15 | 80.5 | 70.8 | 14.4 | 12 | 10.8 | 92 | 98.9 |

Table S4. Clinical data of the second experiment.

| # Subject | Reference HR [bpm] | HR extracted by the optical sensor [bpm] | Reference RR [bpm] | RR extracted by the optical sensor [bpm] | RR extracted by the strain sensor [bpm] | Reference SpO2 [%] | SpO2 extracted by the optical sensor [%] |
| --- | --- | --- | --- | --- | --- | --- | --- |
| 1 | 69 | 64.8 | 12.6 | 12 | 12 | 97 | 99.9 |
| 2 | 86.5 | 90 | 9.6 | 10.8 | 9.6 | 98 | 98.6 |
| 3 | 86 | 87.6 | 21.6 | 20.4 | 19.2 | 97.5 | 99.1 |
| 4 | 82.5 | 79.2 | 15.6 | 13.2 | 13.2 | 99 | 100.1 |
| 5 | 69 | 67.2 | 12 | 10.8 | 9.6 | 97.5 | 99.7 |
| 6 | 63 | 66 | 16.2 | 16.8 | 15.6 | 96 | 99.5 |
| 7 | 79.5 | 69.6 | 30 | 28.8 | 26.4 | 98.5 | 99.2 |
| 8 | 57 | 66 | 20.4 | 22.8 | 12 | 98 | 95.6 |
| 9 | 75.5 | 73.2 | 13.2 | 10.8 | 10.8 | 96.5 | 98.1 |
| 10 | 106.5 | 64.8 | 20.4 | 20.4 | 18 | 98 | 99.8 |
| 11 | 62 | 64.8 | 13.8 | 15.6 | 10.8 | 98 | 99.6 |
| 12 | 69.5 | 62.4 | 7.8 | 14.4 | 9.6 | 93.5 | 99.6 |
| 13 | 72.5 | 72 | 18 | 14.4 | 18 | 97.5 | 99.9 |
| 14 | 57 | 70.8 | 25.2 | 26.4 | 16.8 | 98.5 | 98.4 |
| 15 | 82.5 | 82.8 | 18 | 20.4 | 18 | 99 | 99.1 |
